# An Incidental Finding of Posterior Nutcracker Syndrome: A Case Report

**DOI:** 10.7759/cureus.71205

**Published:** 2024-10-10

**Authors:** Ayman Nadeem, Mohammed Haroon Ahmed, Taneem Ilyas, Mallikarjuna S Oruganti, Saieesh Bairam

**Affiliations:** 1 General Surgery, Osmania Medical College, Hyderabad, IND; 2 Radiodiagnosis, Gandhi Medical College, Hyderabad, IND; 3 Internal medicine, Osmania Medical College, Hyderabad, IND; 4 Internal Medicine, Osmania Medical College, Hyderabad, IND; 5 Radiology, Osmania Medical College, Hyderabad, IND

**Keywords:** abdominal pain, diagnostic challenges, hematuria, posterior nutcracker syndrome, retro-aortic left renal vein compression

## Abstract

Posterior nutcracker syndrome (PNCS) is a rare vascular disorder involving the compression of the retro-aortic left renal vein between the aorta and the vertebral column. It often presents with left flank pain, hematuria, and varicocele.

We report the case of a 39-year-old male who presented with diffuse abdominal pain, acute urinary retention, and left flank pain. Radiologic evaluation revealed retro-aortic left renal vein compression with proximal dilatation, consistent with PNCS.

The atypical presentation and non-specific clinical findings led to a conservative management approach. This case highlights the diagnostic challenges of asymptomatic PNCS and underscores the need for further research into its management.

## Introduction

Posterior nutcracker syndrome (PNCS) is a rare variant of nutcracker syndrome (NCS) characterized by the compression of the left renal vein (LRV) between the aorta and the lumbar vertebrae, leading to venous hypertension. It commonly presents with flank abdominal pain, hematuria, possible varicocele in males, and pelvic congestion in females. The incidence of a retro-aortic LRV is less than 2% [1], and its conjunction with NCS is exceedingly uncommon [2]. This report describes the case of a 39-year-old male with an unusual presentation of PNCS, contributing to the understanding of its varied clinical manifestations.

## Case presentation

A 39-year-old man with a history of chronic alcohol-induced pancreatitis presented with a one-day history of diffuse abdominal pain and shortness of breath. The pain, which was more severe in the epigastric and left hypochondriac regions and radiated to the back, was managed with opioid analgesics. He also experienced acute urinary retention, which was relieved by Foley catheterization. On arrival, his vital signs were as follows: blood pressure 110/80 mm Hg, heart rate 92 beats per minute, and a temperature within normal limits, with an oxygen saturation of 90%. Physical examination revealed diffuse abdominal tenderness and voluntary guarding.

All the laboratory tests, as shown in Table 1 and Table 2 (complete blood count, metabolic panel, viral serology), came back normal, except serum amylase and lipase, which were elevated. An arterial blood gas (ABG) analysis indicated respiratory acidosis, as shown in Table 3.

**Table 1:** Results of the complete blood count

Complete blood count	Result	Reference range
White blood count	12,000/mm^3^	4500-11,000/mm^3^
Red blood count	4.8 million/mm^3^	4.3-5.9 million/mm^3^
Hemoglobin	14.7 g/dL	13.5-17.5 g/dL
Platelets	199,000/mm^3^	150,000-400,000/mm^3^
Mean corpuscular volume	86.4 fL	80-100 fL
Hematocrit	46.5%	41%-53%

**Table 2:** Results of the complete metabolic panel

Complete metabolic panel	Result	Reference range
Sodium	136 mEq/L	136-146 mEq/L
Potassium	3.8 mEq/L	3.5-5 mEq/L
Chloride	103 mEq/L	95-105 mEq/L
Amylase	521 U/L	25-125 U/L
Urea nitrogen	15 mg/dL	7-18 mg/dL
Creatinine	0.6 mg/dL	0.6-1.2 mg/dL
Calcium	8.9 mg/dL	8.4-10.2 mg/dL
Magnesium	1.7 mEq/L	1.5-2 mEq/L
Total protein	5.7 g/dL	6.0-7.8 g/dL
Albumin	3 g/dL	3.5-5.5 g/dL
Alkaline phosphatase	72 U/L	25-100 U/L
Aspartate aminotransferase	19 U/L	12-38 U/L
Alanine aminotransferase	10 U/L	10-40 U/L
Total bilirubin	1.2 mg/dL	0.2-1.1 mg/dL
Lipase	484 U/L	10-140 U/L
Uric acid	4.09 mg/dL	3.0-8.2 mg/dL

**Table 3:** Results of arterial blood gas analysis

Parameter	Result	Reference range
pH	7.291	7.35-7.45
PaCO_2_	52 mm Hg	33-45 mm Hg
PO_2_	83.9 mm Hg	75-105 mm Hg
HCO_3_-	25 mEq/L	22-28 mEq/L

Imaging findings and impression

A clinical diagnosis of recurrent acute pancreatitis with serositis and mild acute respiratory distress syndrome was made. The patient was then referred for further imaging to confirm the diagnosis and assess the severity of pancreatitis. A triphasic contrast-enhanced CT (CECT) scan of the abdomen was performed.

Features suggestive of acute pancreatitis included peripancreatic fat stranding, pancreatic enlargement, and possible fluid collections. The left kidney measured 9.2 x 4.6 cm, normal in size and position. There were a few tiny calculi in the lower pole of the left kidney, with the largest measuring 4 mm. A retro-aortic LRV was noted, with proximal dilatation of the LRV and an aorta-vertebral distance of 3.06 mm. The renal pelvis and calyces were normal, and the urinary drainage tract was unobstructed. The right kidney measured 9 x 4.7 cm, normal in size and position, with normal renal parenchyma, pelvis, and calyces. The urinary drainage tract was unobstructed.

**Figure 1:**
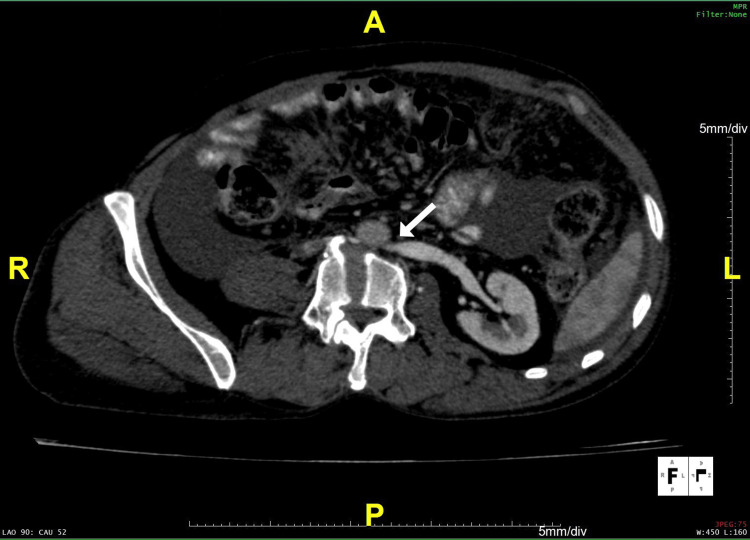
Axial section on contrast-enhanced CT with multi-planar reformation, showing the compression of the left renal vein between the abdominal aorta and vertebral body

**Figure 2:**
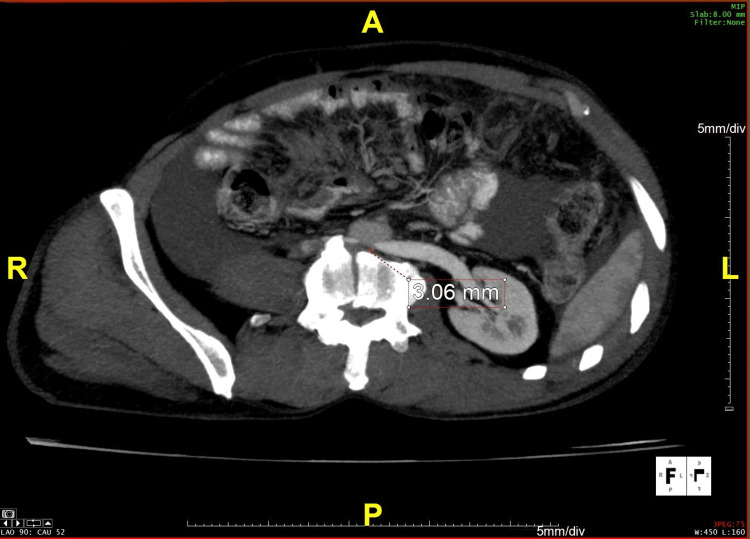
Axial section on contrast-enhanced CT with maximum intensity projection, showing an aorto-vertebral distance of 3.06 mm

Despite these findings (Figures 1, 2), the patient did not exhibit symptoms typically associated with PNCS, such as intermittent hematuria, pelvic pain, left lower back pain, costovertebral angle tenderness, elevated serum creatinine, orthostatic proteinuria, or varicocele. Therefore, the patient was incidentally diagnosed with the posterior nutcracker phenomenon (i.e., PNCS without symptoms). Due to the patient's obesity, a Doppler ultrasound to assess the flow dynamics of the LRV was not feasible.

## Discussion

NCS, first described by pathologist Grant in 1937, involves the compression of the LRV. A study by Poyraz et al. reported a 10.9% prevalence of the nutcracker phenomenon in abdominal computed tomography scans conducted for various reasons [3]. However, the lack of standardized diagnostic criteria and variations in clinical presentation complicate the precise determination of NCS prevalence [4].

NCS is classified into two types: the more common anterior-type NCS occurs when the LRV is compressed between the aorta and the superior mesenteric artery (SMA). In contrast, the rarer posterior-type NCS involves compression of the LRV between the aorta and the vertebral body [5].

Our case involved PNCS presenting with diffuse abdominal pain localized to the epigastric and left hypochondriac regions, which radiated to the back. Additionally, the patient experienced shortness of breath and acute urinary retention. Despite these symptoms, classical hematuria, often associated with NCS, was notably absent. Physical examination revealed voluntary guarding and diffuse tenderness, consistent with acute pancreatitis, a separate condition.

Typically, NCS symptoms include hematuria, orthostatic proteinuria, flank pain, and varicoceles in males or pelvic varices in females. Symptoms can also include pelvic pain, left lower back pain, and systemic manifestations such as headache and tachycardia. The absence of these symptoms in our patient suggested that the presence of PNCS was incidental [6].

A CECT of the abdomen performed to confirm the diagnosis of acute pancreatitis revealed a retro-aortic LRV compressed between the aorta and the vertebral column, with an aorta-vertebral distance of 3.06 mm.

For diagnosing anterior NCS, imaging modalities such as CT and MRI are used to evaluate the aorta-mesenteric angle (less than 25°-41°) and diameter ratios (greater than 2.25-4.9) [7]. Duplex ultrasound with a peak systolic velocity ratio greater than 4.7 between the point of compression and the hilar LRV has shown good sensitivity and specificity [8].

In cases in which the diagnosis of NCS remains ambiguous, further tests such as multidetector CT or MRI with venous phase imaging, renocaval pressure gradients over 3 mmHg, or intravascular ultrasound can be used to confirm compression. Lumbar venography, although invasive, provides definitive confirmation by measuring the pressure gradient between the inferior vena cava and LRV [7].

For PNCS, high-resolution imaging studies should reveal compression of the LRV between the aorta and the vertebral body. However, specific diagnostic cutoff values for this rare type are not well-established [7]. CT can visualize the compression and its hemodynamic consequences, such as dilated gonadal veins and pelvic varices, while MRI provides excellent anatomical detail [6].

Management of our patient focused on pain control with opioid analgesics and addressing acute urinary retention through Foley catheterization. Given the incidental finding of PNCS, treatment concentrated on symptomatic relief and managing acute pancreatitis. Specific interventions for NCS were not indicated but would be guided by symptom severity and patient condition in follow-ups.

Conservative management is generally recommended for younger patients with mild symptoms, including regular follow-ups. For significant symptoms or complications, surgical options such as renal vein bypass, medial nephropexy, transposition with polytetrafluoroethylene, superior mesenteric artery transposition, or Dacron/saphenous vein grafts may be considered. Endovascular stenting is another approach but may be less effective for PNCS due to anatomical challenges. Surgical management is often preferred as the gold standard, with options tailored to the individual’s presentation and response to initial treatments [9].

## Conclusions

In conclusion, this case highlights an incidental finding of PNCS without specific clinical symptoms indicative of retro-aortic LRV compression. Due to the absence of specific diagnostic criteria, management remains conservative, involving continuous monitoring for potential symptom progression and development. This underscores the need for further research to define clear diagnostic criteria and determine the optimal timing for surgical intervention in such cases.
